# Non-response bias in estimates of prevalence of club-based sport participation from an Australian national physical activity, recreation and sport survey

**DOI:** 10.1186/s12889-018-5793-y

**Published:** 2018-07-18

**Authors:** J. T. Harvey, M. J. Charity, N. A. Sawyer, R. M. Eime

**Affiliations:** 1 School of Health and Life Sciences, Federation University Australia, Ballarat, Australia; 2 Institute for Health and Sport, Victoria University, Melbourne, Australia

**Keywords:** Survey non-response bias, Sport, Participation

## Abstract

**Background:**

An estimate of the prevalence of an activity derived from a sample survey is potentially subject to non-response bias, whereby people not involved in the activity are less likely to respond than those involved. Quantifying the extent of non-response bias is generally difficult, since it involves estimating differences between respondents for whom data is directly available from the survey, and non-respondents, for whom data is generally not directly or readily available. However, in the case of the Australian Exercise Recreation and Sport Survey (ERASS), comparative “gold standard” benchmarks exist for some aspects of the survey, in the form of state sporting association (SSA) registration databases, each of which purports to constitute a complete enumeration of club-based players of a particular sport.

**Methods:**

ERASS estimates of the prevalence of participation in four major club-based team sports in the Australian state of Victoria in the year 2010 were compared with prevalences based on numbers of registered participants in the corresponding SSA databases. Comparisons were made for the adult population as a whole (ERASS scope being 15+ years of age), and for strata defined by age and geographical region. Because three of the four sports investigated are strongly sex-specific, no sex breakdowns were conducted. In each case the proportion of ERASS respondents reporting participation, with associated confidence limits, was compared with the corresponding SSA count expressed as a proportion of the population, to form an ERASS/SSA prevalence ratio with associated confidence limits.

**Results:**

The 24 ERASS/SSA ratios ranged from 1.72 to 7.80. Most ratios lay in the range 2 to 3. The lower 95% confidence bound for the ratio was greater than 1.0 in 23 out of 24 cases.

**Conclusions:**

ERASS estimates of prevalence of these particular aspects of sport participation were higher than SSA estimates, to statistically significant degrees. The effect sizes (i.e. the discrepancies represented by the ratios) were large enough to be of great practical importance. It is conjectured that non-response bias is the most likely explanation for the discrepancies.

## Background

As is the case in many other nations, Australia’s population is predominantly inactive and overweight, which has major health implications [1, 2] Data regarding participation in leisure-time physical activity (LTPA), including physical activity (PA) in general, and sport specifically, is important for understanding the effectiveness of different strategies, programs and policies for the promotion of LTPA, and can also be tied to health outcomes of individuals and communities [3].

Population-wide sample surveys are often used for gathering data in this context. However, all such surveys are potentially susceptible to non-response or self-selection bias, whereby the answers of survey respondents differ from the potential answers of those who did not respond. More specifically, estimates of the prevalence of an activity (the proportion of a population or sub-population who engage in the activity) are particularly subject to non-response bias when the propensity towards non-response is related to the propensity towards involvement in the activity under consideration [4]. In surveys of PA, prevalences will be overestimated if persons who are more physically active are more likely to agree to participate in the survey [5]. However, if face-to-face or telephone interviews are conducted at a prime recreational time such as late afternoon, early evening or on weekends, when more active people are less likely to be contactable, it can be conjectured that there may be an opposing tendency towards underestimating prevalences.

Organisations such as the European Survey Research Association (ESRA) established in 2008, and the more specifically focused International Workshop on Household Survey Nonresponse, established in 1990 and since held annually, have documented a steady decline in response rates over two decades [4]. Research into reasons for the decline, strategies to halt or reverse the decline, and assessment of the effects of the decline on accuracy of estimates have also been reported [6].

Estimating the accuracy of estimates, i.e. the extent of bias, is an inherently difficult task, since it involves estimating differences between respondents for whom data is directly available from the survey, and non-respondents, for whom data is not directly or readily available. Lynn [7] presented a summary of possible sources of information about non-respondents. These included: the sampling frame, particularly when sampling from administrative records; geographical information such as postal/zip codes which can be linked to census and other sources regarding demographic characteristics of small areas; interviewer reports; follow-up surveys of non-respondents; and modelling, for example imputing the responses of ‘hard to contact’ respondents to non-respondents. Lynn also advocated the use of weighting for non-response in order to reduce bias, distinguishing between sample-based weights, whereby a set of classes with different response rates is identified and each class is reciprocally weighted according to its response rate, and the more commonly used population-based weights, or post-stratification [8], with weighting classes based on variables which are known both for respondents (from the survey data) and for the population as a whole (from a source such as a census), and with weights being assigned in proportion to the ratio of population to achieved sample in each class. Holt and Elliot [9] presented a more technical exposition of methods of non-response weighting, and in particular drew an important conceptual distinction between two components of bias: differences between the response rates of different weighting classes, and differences in the responses of respondents and non-respondents within each weighting class.

In line with the modelling option of Lynn [7], some researchers have sidestepped the issue of non-response per se, and instead investigated the less difficult question of the differences between the responses of ‘easy to reach’ and ‘difficult to reach’ respondents. Using data from the Canadian Physical Activity Monitor (PAM), a household survey of PA, Craig et al. [10] used three related measures to characterize the ease/difficulty of reaching a selected respondent in each household: the number of telephone calls made to the household; the number of calls answered by someone in the household; and the number of contacts with the selected respondent. The third measure arose because initial refusers were contacted on one or more later occasions by more senior interviewers. The researchers coded each measure into a set of ordinal categories and compared the prevalence of an adequate level of PA (defined as at least 5294 kJ or 1260 MET-minutes per week – equivalent to at least 60 min of moderate-intensity activity daily) in each category. No significant differences were found.

In line with the geographical information option of Lynn [7], other researchers have used census summaries of demographic characteristics of small areas to model the responses of non-respondents [6, 7]. For example, Lee et al. [11] and Lim et al. [12] assigned, to both survey respondents and non-respondents, values of proxy demographic predictors based on the demographic characteristics of their ‘neighbourhoods’ (such as zip-code areas and counties) derived from census and other sources. They then used the survey responses of respondents and the proxy demographic measures for their neighbourhoods to build multilevel logistic regression models for predicting survey responses from small-area demographic characteristics, with the additional incorporation of random small-area effects to allow for the possible effects of geographic clustering. The fitted multilevel logistic regression models were then used to project the survey responses of both respondents and non-respondents (i.e. the whole population) based on the characteristics of the neighbourhoods of both samples. Non-response bias was estimated by comparing these projected survey responses for the whole population with the observed survey responses of the respondent sample. The Lim et al. [12] study was based on data from the 2008 New York City Community Health Survey, a landline telephone survey of residential households in New York, which included the following PA question: “During the past 30 days, other than your regular job, did you participate in any physical activities or exercises such as running, calisthenics, golf, gardening, or walking for exercise?”. The prevalences of a “yes” response were 72.7% (observed respondents), 72.6% (projected respondents), 74.1% (projected non-respondents), 73.7% (projected whole population), with an estimated non-response bias of (72.7–73.7) = − 1.0%, indicating that the survey estimate of prevalence was biased downwards by 1.0 percentage point [12].

A major national survey of recreational PA in Australia, the Exercise, Recreation and Sport Survey (ERASS) commissioned by the Australian Sports Commission (ASC), was conducted annually throughout the period 2001–2010. In 2006, the ASC made a submission regarding “a perception that ERASS is overstating participation in exercise, recreation and sport” to an Inquiry into women in sport and recreation in Australia being conducted at the time by the Environment, Communications, Information Technology and the Arts Reference Committee of the Senate of the Parliament of Australia [13]. According to the ASC submission, the perception of bias had arisen because the 2002 ERASS results reported an overall participation rate 15.4 percentage points higher than the General Social Survey (GSS) conducted by the Australian Bureau of Statistics [14, 15] in the same year (77.8% compared to 62.4%). Consequently, ASC had commissioned a commercial market research company to investigate the possible reasons for the discrepancy. The conclusion reported was that ERASS and the GSS were, broadly speaking, measuring different concepts, specifically that GSS was measuring a narrower concept of PA than ERASS. The GSS questioning approach reportedly used the terms “participant”, “coach”, “official”, “umpire” and “administrator” in the preamble to its key question, which it was argued resulted in GSS respondents being less likely than ERASS respondents to include recreational physical activities like walking, aerobics and yoga in their survey responses.

A further finding was that even though the GSS had a higher response rate than ERASS (91% compared to 46% in 2002), this had not biased ERASS results. This finding was based on the results of a survey conducted using a split sample design, where 1400 respondents were surveyed using Computer-Assisted Telephone Interviewing (CATI). A randomly selected half of the sample (700 respondents) were asked the ERASS question, while the other half (700 respondents) were asked the GSS question. Even though this study achieved a response rate of just 32%, the participation rates obtained from the GSS and ERASS questions in this study were reported to be comparable to the results of the original surveys. It was concluded that there was no evidence of bias in the ERASS data caused by response rates.

More recently, a unique opportunity has arisen for a more direct investigation of non-response bias in ERASS. The Sport and Recreation Spatial project (SRS) www.sportandrecreationspatial.com.au [16], conducted by a research team based at Federation University Australia and Victoria University in the Australian state of Victoria, has entered into partnerships with a number (11 in 2016) of Victorian state sporting associations (SSAs), to integrate and analyse their databases of registered participants, for the purpose of providing an improved information resource for evidence-based planning and policy development across the Victorian sport industry, government and health sectors. Four SSAs, responsible for four of the most popular team sports, have provided SRS with retrospective registration data for the year 2010, the last year in which ERASS was conducted. A condition of the data being provided was anonymity, and so they are referred to throughout this paper as Sports A, B, C and D. To the extent that club-based players can be assumed to be registered at state level, these data purport to constitute a complete enumeration of participants in club competitions sanctioned by the SSAs of these four sports in Victoria, against which corresponding ERASS estimates of club-based participation can be directly compared.

Hence the aim of this study was to compare 2010 ERASS estimates of club-based participation in four sports in Victoria with corresponding 2010 SSA counts of registered participants, for the population of participants as a whole, and for population subgroups defined by age and geographical regions.

## Methods

### ERASS survey data

The ERASS survey entailed a series of independent cross-sectional national surveys conducted yearly between 2001 and 2010, with the aim of collecting information on the recreational physical activities of Australians [17, 18]. The usefulness of ERASS for public health surveillance has been previously reported [3], and various studies have drawn from ERASS data to describe, for example, participation trends of leisure-time PA [3], the diversity of physical activities engaged in by older people [19] and population percentages meeting muscle-strengthening activity guidelines [20].

ERASS comprised a random survey stratified by state and territory, aimed at persons aged 15 years and over residing in occupied private dwellings. Using a CATI system, data were collected quarterly, with households being sampled from the Electronic White Pages (2001–2006), by random digit dialing (2007–2009), and for the data used in this study (2010), from a marketing and social research industry database called SamplePages, which provided coverage of both fixed landlines and mobile phones [18]. On being contacted by telephone, one person per dwelling was randomly selected (most recent birthday method) to complete the interview. Respondents were informed about the purpose and background of the ERASS, assured of confidentiality of the data and were given the opportunity to ask questions. Verbal informed consent was indicated by the respondents’ willingness to participate in the telephone survey.

ERASS data for the state of Victoria collected in the year 2010 were analysed for the present study. The data used in this study comprised sex, age, residential postcode (and hence region – ‘capital city’ or ‘rest of state’), and data about club-based sport participation.

ERASS respondents were first asked whether they had participated in any PA for exercise, recreation or sport during the 12 months prior to the date they were surveyed. Those who had done so were asked to nominate up to 10 types of PA from a classification of 95 types (e.g. basketball, tennis, aerobics, walking). This classification included both sports, defined in Australia as a PA that has a sport governing body and by its nature and organisation is competitive and is generally accepted as being a sport [21], and other forms of recreational PA. Hence, for each of the 95 ERASS PA types, each respondent could be classified as a participant or a non-participant. For each PA type nominated, participants were asked “Was any of this (activity) organised by a club, association or other type of organisation?” If they answered yes, they were then asked to indicate any of the following five organisational settings which applied to them: 1) fitness, leisure or indoor sports centre that required payment for participation; 2) sport or recreation club or association that required payment of membership, fees or registration; 3) work; 4) school; and 5) other (specify). In accordance with the aims of the present study, for each of the four sports included in the study, the target group consisted of all persons who indicated option 2 “sport or recreation club or association that required payment of membership, fees or registration” for that sport, regardless of whether they also participated in other settings. For brevity, for each sport this group is referred to throughout as “club participants”. For each sport, all other respondents to the survey were classified as not being club participants for that sport.

### Sport participant registration data

This study also drew on the participant registration records from four club-based team sports in the state of Victoria for the year 2010. A club participant was defined as a participant registered in 2010 with the respective sport’s SSA.

Available data regarding each participant included: sport, date of birth, sex and residential postcode (which enabled the assignment of each player to a geographical region – either ‘capital city’ or ‘rest of state’, as for the ERASS data). A ‘census’ date for each sport (1 January for three of the sports and 1 July for one sport) was used to determine each participant’s age in years in the 2010 competition season. Consistent with ERASS, the data were limited to registered participants aged 15 years and over in 2010. Participants for whom age or residential postcode were unknown were excluded from the analysis. The percentage of participants excluded for each sport were: A 1.0%; B 17.0%; C 1.9%; and D 1.2%.

### Method of analysis

Because the ERASS was limited to persons aged 15 years and over, all references in the following to totals for Australia, Victoria, and capital city and rest of state regions apply to persons aged 15 years and over.

For each of the four sports studied, ERASS and SSA estimates of the number of club-based participants in the state of Victoria (aged 15 years and over) were calculated, and also broken down by region (capital city v rest of state) and three age ranges (15–19 years, 20–49 years, 50 years and over). Because three of the four sports are highly sex-specific, breakdowns by sex were not conducted.

The two estimated counts for each geographic and age category were thus:

- N1: The number of registered participants in the SSA dataset.
- N2: The number of club participants estimated from the ERASS data.

ERASS estimates were calculated using a weighted analysis. The weights provided with ERASS data are population-based, using estimated resident population (ERP) in each “cell” of a 4-factor classification: state/territory × region (capital city, rest of state/territory) × sex × age (6 categories). ERASS weights have two constituent components which serve different purposes. The first component is designed to make the contribution of the sample data from each cell (to sample estimates of population parameters) proportional to the corresponding segment of the population in each cell. To achieve this, each response in under-represented cells must be given more weight and each response in over-represented cells must be given less weight. Responses from persons in cells which are under-represented in the ERASS sample (relative to the population) are up-weighted (multiplied by a weight > 1) and responses from cells which are over-represented in the ERASS sample are down-weighted (multiplied by a weight < 1). In the resulting weighted estimates of population parameters each person in the population is considered to be equally represented. These “primary” sample weights sum to the total sample size for the whole of Australia. Second, the sample weights are rescaled (or “grossed up”) by multiplying each weight by the ratio of the total Australian population (aged 15 years and over) to the total sample size. This rescaling results in weights which not only sum to the total population of Australia (aged 15 years and over), they also sum to the population counts in each cell of the cross-classification. Consequently, weighted sample estimates of the numbers of persons with a particular characteristic (such as playing a particular sport) are direct estimates for the population or relevant sub-population. Finally, ERASS weights are divided by 1000, so that the weighted estimates represent counts expressed in thousands.

Because N1 purports to be a complete enumeration of registered participants, it contains no sampling error. The same is true of the estimated resident population. For this reason, statistical inference was limited to calculating confidence limits for N2, based on standard errors estimated using the SPSS Complex Samples procedure. Confidence limits for the participation rate N2/ERP, where ERP is the corresponding estimated resident population, and for the ratio R = N2/N1, follow by simple proportional rescaling of the confidence limits of N2. Of course, N1 is subject to non-sampling errors due to imperfections in the SSA registration systems. The numbers of registered participants in each sport who were excluded from the analysis because of missing data regarding age, sex or residential postcode are known, but the number of participants who were not registered cannot be quantified.

Ethics approval was granted by the Human Research Ethics Committee of the Federation University, Australia (Ref C13–007).

## Results

Table 1 shows the results for the four sports, first for all Victorians aged 15 years and over (the limit of ERASS), and then for breakdowns into capital city (i.e. Melbourne) and Rest of Victoria regions, and three age cohorts. The final result in each case is the ratio of the ERASS estimate (of the number or the participation rate) to the corresponding number or registration rate from the SSA data. The 24 ERASS/SSA ratios range from 1.72 to 7.80. Most ratios lie in the range 2 to 3. It is noticeable that for each of the four sports the ratio for the oldest age group (50 years and over) stands out as being much higher than all the other ratios.

**Table 1:** Comparison of prevalences of club sport participation based on ERASS^a^ and SSA^b^ registration data

	Sport A	Sport B	Sport C	Sport D
Victoria (aged 15 years and over)				
ERASS				
Sample size	6073	6073	6073	6073
Reported club participants in sample	100	87	135	102
Population (ERP 15+)^c^	4,376,239	4,376,239	4,376,239	4,376,239
Estimated club participants^d^	98,656	95,760	154,239	103,946
Standard error of estimate^e^	10,510	11,531	14,807	11,024
95% confidence limit - lower	78,056	73,159	125,217	82,338
95% confidence limit - upper	119,255	118,362	183,260	125,553
Estimated participation rate	0.0225	0.0219	0.0352	0.0238
95% confidence limit - lower	0.0178	0.0167	0.0286	0.0188
95% confidence limit - upper	0.0273	0.0270	0.0419	0.0287
SSA				
Registered club participants	47,301	33,760	61,559	44,663
Registration rate	0.0108	0.0077	0.0141	0.0102
Comparison				
Ratio (ERASS/SSA)	2.09	2.84	2.51	2.33
95% confidence limit - lower	1.65	2.17	2.03	1.84
95% confidence limit - upper	2.52	3.51	2.98	2.81
Melbourne				
ERASS				
Sample size	4302	4302	4302	4302
Reported club participants in sample	49	68	87	69
Population (ERP 15+)^c^	3197,110	3197,110	3197,110	3197,110
Estimated club participants^d^	49,596	71,078	90,293	69,453
Standard error of estimate^e^	7478	9208	10,309	9044
95% confidence limit - lower	34,938	53,030	70,087	51,727
95% confidence limit - upper	64,254	89,126	110,499	87,178
Estimated participation rate	0.0155	0.0222	0.0282	0.0217
95% confidence limit - lower	0.0109	0.0166	0.0219	0.0162
95% confidence limit - upper	0.0201	0.0279	0.0346	0.0273
SSA				
Registered club participants	25,269	25,426	34,393	30,458
Registration rate	0.0079	0.0080	0.0108	0.0095
Comparison				
Ratio (ERASS/SSA)	1.96	2.80	2.63	2.28
95% confidence limit - lower	1.38	2.09	2.04	1.70
95% confidence limit - upper	2.54	3.51	3.21	2.86
Rest of Victoria				
ERASS				
Sample size	1771	1771	1771	1771
Reported club participants in sample	51	19	48	33
Population (ERP 15+)^c^	1,179,128	1,179,128	1,179,128	1,179,128
Estimated club participants^d^	49,060	24,682	63,946	34,493
Standard error of estimate^e^	7438	6983	10,718	6366
95% confidence limit - lower	34,480	10,995	42,939	22,015
95% confidence limit - upper	63,639	38,368	84,953	46,971
Estimated participation rate	0.0416	0.0209	0.0542	0.0293
95% confidence limit - lower	0.0292	0.0093	0.0364	0.0187
95% confidence limit - upper	0.0540	0.0325	0.0720	0.0398
SSA				
Registered club participants	22,032	8334	27,166	14,205
Registration rate	0.0187	0.0071	0.0230	0.0120
Comparison				
Ratio (ERASS/SSA)	2.23	2.96	2.35	2.43
95% confidence limit - lower	1.57	1.32	1.58	1.55
95% confidence limit - upper	2.89	4.60	3.13	3.31
Aged 15–19 years				
ERASS				
Sample size	378	378	378	378
Reported club participants in sample	36	29	54	29
Population (ERP 15+)^c^	470,994	470,994	470,994	470,994
Estimated club participants^d^	44,588	37,842	61,757	33,272
Standard error of estimate^e^	7529	8120	9158	6312
95% confidence limit - lower	29,831	21,927	43,808	20,900
95% confidence limit - upper	59,345	53,756	79,706	45,644
Estimated participation rate	0.0947	0.0803	0.1311	0.0706
95% confidence limit - lower	0.0633	0.0466	0.0930	0.0444
95% confidence limit - upper	0.1260	0.1141	0.1692	0.0969
SSA				
Registered club participants	17,774	13,100	25,185	12,291
Registration rate	0.0377	0.0278	0.0535	0.0261
Comparison				
Ratio (ERASS/SSA)	2.51	2.89	2.45	2.71
95% confidence limit - lower	1.68	1.67	1.74	1.70
95% confidence limit - upper	3.34	4.10	3.16	3.71
Aged 20–49 years				
ERASS				
Sample size	2350	2350	2350	2350
Reported club participants in sample	54	51	75	56
Population (ERP 15+)^c^	2,170,864	2,170,864	2,170,864	2,170,864
Estimated club participants^d^	48,263	54,165	88,661	59,890
Standard error of estimate^e^	7137	8100	11,612	8671
95% confidence limit - lower	34,275	38,289	65,901	42,895
95% confidence limit - upper	62,251	70,041	111,422	76,886
Estimated participation rate	0.0222	0.0250	0.0408	0.0276
95% confidence limit - lower	0.0158	0.0176	0.0304	0.0198
95% confidence limit - upper	0.0287	0.0323	0.0513	0.0354
SSA				
Registered club participants	28,125	20,179	35,826	30,109
Registration rate	0.0130	0.0093	0.0165	0.0139
Comparison				
Ratio (ERASS/SSA)	1.72	2.68	2.47	1.99
95% confidence limit - lower	1.22	1.90	1.84	1.42
95% confidence limit - upper	2.21	3.47	3.11	2.55
Aged 50 years and over				
ERASS				
Sample size	3197	3197	3197	3197
Reported club participants in sample	10	7	5	17
Population (ERP 15+)^c^	1,647,164	1,647,164	1,647,164	1,647,164
Estimated club participants^d^	5804	3754	3218	10,783
Standard error of estimate^e^	1924	1487	1458	2733
95% confidence limit - lower	2033	840	360	5427
95% confidence limit - upper	9576	6667	6076	16,139
Estimated participation rate	0.0035	0.0023	0.0020	0.0065
95% confidence limit - lower	0.0012	0.0005	0.0002	0.0033
95% confidence limit - upper	0.0058	0.0040	0.0037	0.0098
SSA				
Registered club participants	1402	481	548	2263
Registration rate	0.0009	0.0003	0.0003	0.0014
Comparison				
Ratio (ERASS/SSA)	4.14	7.80	5.87	4.76
95% confidence limit - lower	1.45	1.75	0.66	2.40
95% confidence limit - upper	6.83	13.86	11.09	7.13

^a^ ERASS: Exercise, Recreation and Sport Survey

^b^ SSA: State sporting association

^c^ Estimated resident population, 2010, as used in ERASS weighting

^d^ Weighted estimate

^e^ Standard error calculated using SPSS Complex Samples analysis

A corresponding set of unweighted analyses was also conducted. The pattern of results (not tabulated) was broadly similar. However, the discrepancies were consistently smaller in magnitude, with ratios ranging from 1.12 to 7.50; in 20 of the 24 cases, the ERASS/SSA ratio was greater for the weighted analysis than for the unweighted analysis. The ratio of weighted/unweighted ratios ranged from 0.92 to 1.57, with a mean value of 1.31.

## Discussion

Whilst surveys are often used to capture information about individuals’ physical activity behaviors, it is imperative that this information truly reflects behaviors, and that the results on which recommendations are made are not based upon biased samples. This study uniquely investigated the possibility of non-response bias in estimates of prevalence of club-based participation in four types of sport from a national survey, by comparing the survey-based estimates with corresponding enumerations of registered participants from the registration databases of each sport.

In summary, relative to the registration counts, the survey sample massively overestimated club-based participation in each of the four sports. In all of the 24 cases examined (4 sports × 6 population segments), the ratio of the ERASS club-based participation estimate to the SSA registration figure was greater than 1.0. The ratios ranged from 1.72 to 7.80. Most ratios lay in the range 2 to 3. Given that the SSA registration counts purport to represent complete counts of club participants (albeit with some known exclusions because of missing data), this is strong evidence that the 2010 ERASS data grossly over-estimated the participation of Victorians in these four sports.

Two potential explanations for these discrepancies are the CATI data collection mode of the ERASS interviews, and non-response bias. The ERASS 2010 Methodology Report included the results of an investigation into the potential for bias due to limitations in the sampling frame of telephone numbers, particularly regarding the absence of mobile phones and silent/unlisted numbers, and concluded that participation in PA was unlikely to be correlated with the type of telephone number.

With regard to non-response bias, it is conjectured that sport participants might be more likely to agree to be interviewed for the ERASS than non-participants. The overall response rate of the 2010 ERASS was reported as 23.1% (or 26.8% when selected numbers for which there was no answer after four calls were excluded) [18]. The corresponding direct refusal rates were 51.1 and 59.2%, or equivalently, non-refusal (or self-selection) rates of 48.9 and 40.8% [18]. Interestingly, if the non-refusal rates are expressed as proportions (0.489 and 0.408), their reciprocals are 2.04 and 2.45. This suggests that if all non-refusers were sport participants and all refusers were not, extrapolating the evidence from the sample of completed interviews to the whole population would tend to over-estimate sport participation by a factor of 2–2.5. This is very close to range of most of the calculated ERASS/SSA ratios, suggesting that non-response bias is the major contributing factor to the discrepancy.

Table 1 also shows that, for all four sports, the discrepancy was greatest for those aged 50 years and over. The greater discrepancy in this age cohort suggests greater self-selection bias, i.e. that the association between sport participation and willingness to participate in the ERASS survey is strongest among this older cohort.

Turning to the results of the unweighted analysis of ERASS data, in 20 of the 24 cases, the ERASS/SSA ratio for the weighted analysis was greater than for the unweighted analysis. The ratio of weighted/unweighted ratios ranged from 0.92 to 1.57, with a mean value of 1.31. This suggests that, rather than reducing the effects of non-response bias, weighting actually tends to exacerbate the over-estimation effect of non-response bias on estimates of participation numbers and rates. It is conjectured that this is because the population-based ERASS weighting (with respect to state, region, sex and age) is not specifically targeted at non-response per se. A feasible scenario is that non-response bias is negatively correlated with response rate, i.e. in those cells where response rates are lower than average, the degree to which responders are more likely to be sport participants than are non-responders is higher than average, and in those cells where response rates are higher than average, the degree to which responders are more likely to be sport participants than are non-responders is lower than average. Under this scenario, population-based weighting increases the contribution (to population estimates) of responders in cells with low response rates (and high non-response bias) and decreases the contribution of responders in cells with high response rates (and low non-response bias), thereby amplifying the effects of non-response bias. This result is consistent with the theoretical analysis of Holt and Elliot [9], who showed that population-based weighting would increase bias under this negative correlation scenario. However, because no information was available about strata-specific response rates for ERASS, it was not possible to investigate this conjecture empirically.

Lynn [7] advocated the use of “sample-based weighting” in order to reduce bias. In sample-based weighting, a set of classes with different response rates is identified and each class is reciprocally weighted according to its response rate. The more commonly used “population-based weighting” seeks to reduce the effects of the achieved sample being unrepresentative of the population profile (in the case of ERASS, with respect to state, region, sex and age). The two approaches both seek to compensate for non-response due to direct refusal, but the latter also seeks to compensate for other factors including completeness of the sampling frame, departures of the profile of the selected target sample from the profile of the population, and contactability of the selected sample. It would have been interesting to repeat the present analysis with sample-based weights, but this was not possible; population-based weights were provided with the ERASS data, but the information required to calculate sample-based weights (i.e. the response rate in each cell) was not available to the authors.

In their recent review of studies of physical activity prevalence in Australian children and adolescents, Pedisic et al. [23] have asked “Why do different surveys provide so different estimates?” The Pedisic et al. study incorporated results from 21 population surveys conducted in Australia during the period 2004–2014, including the extension to the 2010 ERASS which covered children and adolescents aged 5–14 years [22]. It is interesting that, of the 18 of these surveys which included adolescents, ERASS was one of three surveys which produced much higher estimates of prevalence than the other 15 surveys, by a factor of around 2.5 - around 50% compared to an average of around 20% for the other 15 surveys. This comparative margin is very similar in magnitude to the comparative margins observed in the present study of older adolescents and adults.

We posit a slightly different question to that of Pedisic et al. [23]: why are the results of the present study so different from the results of the previously reported investigation into bias in ERASS estimates? In 2006, the ASC reported on an investigation into potential bias in the overall ERASS estimate of the PA participation rate (77.8%) compared to that derived from the General Social Survey (GSS) conducted by the Australian Bureau of Statistics [14, 15](62.4%). The conclusion reported was that ERASS and the GSS were, broadly speaking, measuring different concepts, specifically that GSS was measuring a narrower concept of PA than ERASS. Results of a specially commissioned study were also reported, in which a randomly selected half of the study sample of 1400 respondents were asked the ERASS question, while the other half were asked the GSS question. Even though this study achieved a response rate of just 32%, the participation rates obtained from the GSS and ERASS questions in this study were reported to be comparable to the results of the original surveys, and it was concluded that there was no evidence of bias in the ERASS data (relative to the GSS data) caused by response rates.

However, considering the low response rate in that study, the results are open to the interpretation that the two estimates based on ERASS and GSS questions were equally biased by non-response. By contrast, in the present study, apart from some differences in time frames (see Limitations below), the conceptual basis of sports participation in ERASS and SSA data were very well aligned, and crucially, the SSA benchmarks against which ERASS estimates were compared purported to be complete enumerations (albeit with some known exclusions due to missing data), and as such were far less subject to non-response bias. However, it is also acknowledged that, given its focus on four club-based sports, the present study was limited in scope to very specific and relatively intensive types of PA, for which survey non-response bias may be greater than for PA in general.

Finally, it must be stressed that the presence of non-response bias in ERASS-based estimates of prevalence does not necessarily invalidate the conclusions of the many comparative analyses that have been conducted over a 15-year period using ERASS data. Conclusions regarding trends over time, cross-sectional comparisons between indicators (such as different types or intensities of activity) or population subgroups (regions based on indicators of socio-economic status or remoteness, local government areas), or correlations between measures (such as age and frequency of PA sessions), are valid to the extent that any bias is consistent across the scope of the data analysed. Having said that, the ERASS response rate was halved (from around 50% to around 25%) and the refusal rate increased by half (from around 33% to around 50%) between 2000 and 2010 [18]. This may have also increased the non-response bias over time, which would threaten the validity of analyses of long-term trends in participation.

The fortuitous circumstance, that ERASS and SSA data for the same epoch became available, enabled the study reported in this paper to be conducted. The fact that the overlap was only for a single year precluded examination of trends in bias over time. However, with the recent establishment of a successor to ERASS, the AusPlay survey [24], and the progressive annual additions and expansions to scope of the repository of SSA datasets, extensions to this research will be possible in future years.

### Limitations

Limitations of this study can be discussed with regard to five aspects of the scope and alignment of the two datasets used in this study: 1) types of sport; 2) geographical scope; 3) temporal scope; 4) organisational settings; and 5) data coverage.

The scope of this study was limited to SSA-affiliated club-based participation by registered participants in four major sports in the Australian state of Victoria. Caution should be exercised in generalising the results beyond that scope. As outlined under methodology, the geographical alignment of the two datasets was good.

With regard to temporal scope and alignment, the time periods relating to SSA and ERASS data were a little different conceptually and a little misaligned. Because data from each ERASS respondent pertained to the 12-month period prior to the survey date, 2010 ERASS data (collected in the four quarters of 2010) collectively pertained to a period of almost 24 months, from early to 2009 to late 2010. Each SSA registration for 2010 pertains to a 12-month period, from January 2010 to December 2010 for three sports, and from July 2010 to June 2011 for one sport. ERASS data from quarters 3 and 4 of 2010 and quarters 1 and 2 from 2011 would have provided a more balanced alignment with calendar year 2010 (24 months centred on 2010 - from mid-2009 to mid-2011), but ERASS ceased at the end of 2010. Conversely, no SSA data prior to 2010 were available to the study. The temporal overlap was fortuitous, but imperfect. Notwithstanding all of this, the timeframe for each individual in both ERASS and SSA contexts was a period of 12-months, at least part of which fell within calendar year 2010. Consequently, it is contended that the inexactitude of the temporal matching of the datasets does not invalidate the conclusions reached to any substantial degree.

Regarding scope and alignment of organisational settings, the study is predicated on the equivalence of, on the one hand, the ERASS concept of participation organised by a “sport or recreation club or association that required payment of membership, fees or registration”, and on the other hand, the concept of “participant registered with an SSA”. While we contend that the alignment is close, we acknowledge that the ERASS response could include a broader class of activities than those of registered SSA participants, including “one-off come-and-try” types of activity at SSA-affiliated clubs, more regular participation at such clubs by players who are not registered with the SSA, and participation through social clubs not affiliated with SSAs. However, given that two of the other organisational setting options (“work” and “school”) would be the most likely contexts of such participation, arguably these would be more likely to be perceived by the casual or social participant as the organisation which organised the activity, even if the activity took place at a sports club venue. Consequently, we think that conceptual misalignment is unlikely to be a major contributing factor to the large discrepancies reported in this study.

Regarding data coverage, our primary contention is that self-selection operates to bias ERASS coverage towards more physically active persons, leading to over-estimates of participation. An alternative explanation is under-representation of club-based participation in SSA registration records, i.e. the converse of over-estimation in ERASS data. There have been anecdotal suggestions of shortcomings in SSA databases, due to poor communication between organisational levels and poor control of “bottom-up” registration processes in some sports, which may have resulted in some participants playing in club settings in some circumstances without necessarily being registered at state level. While some registered participants were excluded from this study because of missing or invalid data in SSA databases regarding age or geographical location, the authors are not aware of objective evidence of “lack of coverage” problems on a scale that would explain the large discrepancies reported in this study. Furthermore, the magnitude and pattern of discrepancies is quite similar across the four sports. Lack of coverage issues would not be expected to be uniform across four sports, whereas non-response bias would be expected to produce similar discrepancies for all sports.

## Conclusion

The possibility of non-response bias is a perennial issue for all survey-based research. The establishment of the Sport and Recreation Spatial project (SRS) and its repository of Victorian SSA databases of registered participants, has provided a unique opportunity for a direct investigation of non-response bias in ERASS. This study compared 2010 ERASS estimates of club-based participation in four sports in Victoria with corresponding 2010 SSA counts of registered participants, for Victorians aged 15 years and over and for population subgroups defined by age and geographical regions.

The 2010 ERASS survey data were found to be subject to considerable non-response bias and to massively over-estimate the prevalences of club-based participation in four major sports. The extent to which this over-estimation may apply to other sports, other forms of PA, other states, and to estimates of PA prevalence from surveys other than ERASS, such as the Australian Bureau of Statistics (ABS) GSS, is not known.

It is stressed that the existence of non-response bias in ERASS-based estimates of prevalence does not necessarily invalidate the conclusions of the many comparative analyses that have been conducted over a 15-year period using ERASS data. Conclusions about trends, cross-sectional comparisons or correlations are valid to the extent that the non-response bias is consistent across the scope of the data analysed.

However, our essential conclusion remains that absolute population counts and prevalences are substantially over-estimated in the ERASS data. Monitoring the prevalence of sport participation over time is a crucial activity to support evidence-based evaluation of sports policies and programs, and for rational planning of future facilities and programs. Furthermore, from a public health perspective, accurate information about the prevalence of health-promoting sporting activities is essential. We contend that for these purposes, population surveys like ERASS cannot provide an adequate substitute for the ongoing study of the registration records of state and national sporting organisations undertaken through activities such as the SRS project. Two other benefits of such data collections are the cost-efficiency of establishing and maintaining them, and their sheer size, which provides a statistically powerful basis for disaggregation and comparative analysis of participation for much smaller geographical regions than is possible with even the largest national sample surveys.

Looking to the future, SRS is uniquely placed to undertake a similar study regarding the newly established AusPlay survey, when the relevant AusPlay data become available.

## Abbreviations

ABS: Australian Bureau of Statistics
ASC: Australian Sports Commission
CATI: Computer-assisted telephone interviewing
ERASS: Exercise, Recreation and Sport Survey
ERP: Estimated resident population
ESRA: European Survey Research Association
GSS: General Social Survey
LTPA: Leisure-time physical activity
PA: Physical activity
SRS: Sport and Recreation Spatial
SSA: State sporting association
